# Draft Genome Sequence of Lactobacillus salivarius KZ-NCB, Isolated from Chicken Cecum

**DOI:** 10.1128/MRA.01129-20

**Published:** 2020-12-10

**Authors:** Saveliy Kirillov, Asset Daniyarov, Aigerim Turgimbayeva, Yerlan Ramankulov, Ruslan Kalendar, Sailau Abeldenov

**Affiliations:** a National Center for Biotechnology, Nur-Sultan, Kazakhstan; b School of Science and Humanities, Nazarbayev University, Nur-Sultan, Kazakhstan; с Department of Agricultural Sciences, University of Helsinki, Helsinki, Finland; Portland State University

## Abstract

Here, we report the draft genome sequence of Lactobacillus salivarius strain KZ-NCB, which was isolated from the cecum of a healthy chicken from a poultry farm in Kazakhstan.

## ANNOUNCEMENT

*Lactobacillus* represents some of the most promising probiotics that have been widely introduced into agriculture, including poultry. Probiotics could be an alternative to feed antibiotics, which for a long time were considered uncontested in the poultry industry (1). The use of feed antibiotics in agriculture is one of the sources of the emergence of antibiotic-resistant strains, which has been recognized by the World Health Organization as one of the 10 greatest threats to global health (2, 3).

The Lactobacillus salivarius KZ-NCB strain was isolated from a 42-day-old Arbor Acres broiler from a local poultry farm (51°03′38.4″N, 70°58′45.5″E). A 10% suspension of cecal contents was inoculated onto MRS agar and cultured at 37°C for 24 h under aerobic conditions, and single colonies were isolated. The Lactobacillus salivarius KZ-NCB strain was cultivated on MRS broth at 37°C for 24 h. The genus and species were identified by 16S rRNA gene sequencing using the BigDye terminator v3.1 cycle sequencing kit on an ABI 3730xl DNA analyzer (Applied Biosystems) with 8F and 806R primers (4), with the strain being closely related to the Lactobacillus salivarius JCM1046 strain, and were confirmed by the matrix-assisted laser desorption ionization (MALDI) Biotyper microbial identification system (Bruker).

Lactobacillus salivarius KZ-NCB was cultured in MRS broth at 37°C for 24 h under aerobic conditions. DNA for whole-genome sequencing was isolated using the QIAamp DNA minikit (Qiagen). The DNA library was prepared using the Nextera XT DNA library preparation kit (Illumina, Inc.) according to the manufacturer's instructions. Sequencing was performed using the paired-end reagent kit v3 (600 cycles) on the MiSeq system (Illumina, Inc.). A total of 739,128 reads were produced, with an average length of 253 bp. The raw sequencing data were analyzed by FastQC v0.11.9 for quality control purposes (5). Quality-filtered reads were assembled using the SPAdes assembler v3.13.2 under the Unicycler program v0.4.8 (6) with default parameters, giving 92 contigs with a total length of 1,865,738 bp, an average GC content of 32.69%, 56-fold coverage, and an *N*_50_ value of 51,932 bp. Assembly quality was evaluated using QUAST v5.0.2 (7). A main annotation was performed by PGAP v4.12 (8), which resulted in a total of 1,769 coding sequences (CDSs) and 51 RNA genes.

For comprehensive proteomic analysis, an assembled genome was additionally annotated by RAST (9) under the PATRIC service (10). A total of 2,018 CDSs, 64 structural tRNAs, and 7 rRNAs were predicted. The RAST annotation included 426 hypothetical proteins and 1,397 proteins with functional assignments. The proteins with functional assignments included 498 proteins with Enzyme Commission (EC) numbers, 417 proteins with Gene Ontology (GO) assignments (11), and 336 proteins that were mapped to KEGG pathways (12).

**FIG 1:**
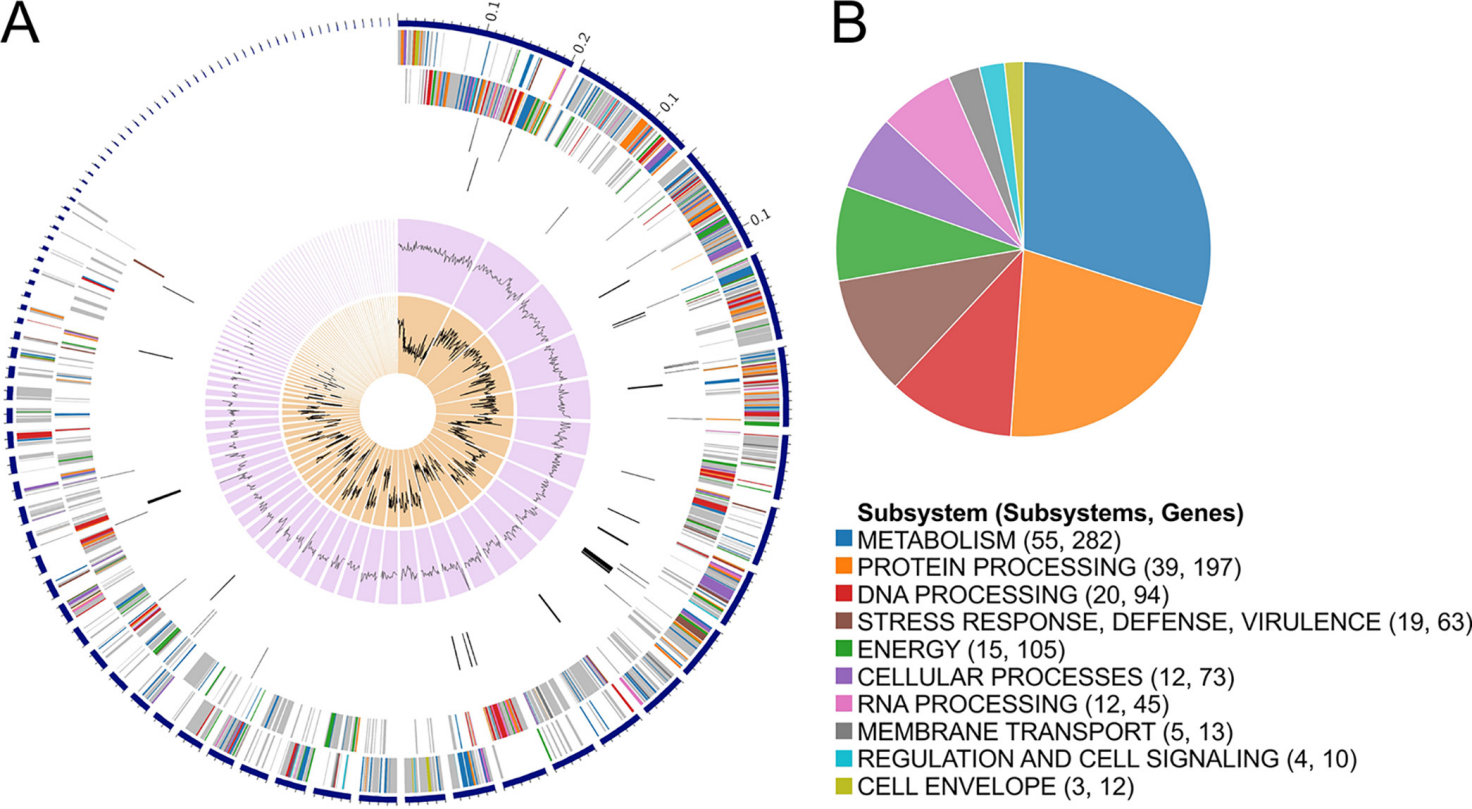
Circular graphical display of the distribution of the genome annotations of Lactobacillus salivarius KZ-NCB. (A) From the outer circle to the inner circle are the contigs, CDSs on the forward strand, CDSs on the reverse strand, RNA genes, CDSs with homology to known antimicrobial resistance genes, CDSs with homology to known virulence factors, GC content, and GC skew (PATRIC was used to generate these data [14, 15]). (B) The colors of the CDSs on the forward and reverse strands indicate the subsystems to which these genes belong.

A circular graphical display of the distribution of the genome annotations is provided in Fig. 1A. A subsystem is a set of proteins that together implement a specific biological process or structural complex (13), and PATRIC annotation includes an analysis of the subsystems unique to each genome (14, 15) (Fig. 1B).

### Data availability.

This whole-genome shotgun project has been deposited in DDBJ/ENA/GenBank under the accession no. JACBGJ000000000. The version described in this paper is the first version, JACBGJ000000000.1. The GenBank assembly accession number is GCA_013391745. The raw data from BioProject no. PRJNA641655 were submitted to the NCBI SRA under accession no. SRX9078425.
